# Predictive Monitoring of Shake Flask Cultures with Online Estimated Growth Models

**DOI:** 10.3390/bioengineering8110177

**Published:** 2021-11-06

**Authors:** Barbara Pretzner, Rüdiger W. Maschke, Claudia Haiderer, Gernot T. John, Christoph Herwig, Peter Sykacek

**Affiliations:** 1 Körber Pharma, Mariahilfer Straße 88A/1/9, 1070 Vienna, Austria; claudia.haiderer@koerber-pharma.com (C.H.); christoph.herwig@tuwien.ac.at (C.H.); 2 Research Area Biochemical Engineering, Vienna University of Technology, Gumpendorfer Strasse 1a, 1060 Vienna, Austria; 3 Institute of Chemistry and Biotechnology, Life Sciences and Facility Management, Campus Grüental, Zurich University of Applied Sciences, 8820 Wädenswil, Switzerland; masc@zhaw.ch; 4 PreSens Precision Sensing GmbH, Am BioPark 11, 93053 Regensburg, Germany; g.john@presens.de; 5 Competence Center CHASE GmbH, Altenbergerstraße 69, 4040 Linz, Austria; 6 Department of Biotechnology, University of Natural Resources and Life Sciences, Vienna, Muthgasse 18, 1190 Vienna, Austria

**Keywords:** particle filter, shake flask, Gompertz function, logistic function, time series forecasting, critical event prediction, harvest time estimation, *Escherichia coli*, strain and substrate optimization

## Abstract

Simplicity renders shake flasks ideal for strain selection and substrate optimization in biotechnology. Uncertainty during initial experiments may, however, cause adverse growth conditions and mislead conclusions. Using growth models for online predictions of future biomass (BM) and the arrival of critical events like low dissolved oxygen (DO) levels or when to harvest is hence important to optimize protocols. Established knowledge that unfavorable metabolites of growing microorganisms interfere with the substrate suggests that growth dynamics and, as a consequence, the growth model parameters may vary in the course of an experiment. Predictive monitoring of shake flask cultures will therefore benefit from estimating growth model parameters in an online and adaptive manner. This paper evaluates a newly developed particle filter (PF) which is specifically tailored to the requirements of biotechnological shake flask experiments. By combining stationary accuracy with fast adaptation to change the proposed PF estimates time-varying growth model parameters from iteratively measured BM and DO sensor signals in an optimal manner. Such proposition of inferring time varying parameters of Gompertz and Logistic growth models is to our best knowledge novel and here for the first time assessed for predictive monitoring of *Escherichia coli* (*E. coli*) shake flask experiments. Assessments that mimic real-time predictions of BM and DO levels under previously untested growth conditions demonstrate the efficacy of the approach. After allowing for an initialization phase where the PF learns appropriate model parameters, we obtain accurate predictions of future BM and DO levels and important temporal characteristics like when to harvest. Statically parameterized growth models that represent the dynamics of a specific setting will in general provide poor characterizations of the dynamics when we change strain or substrate. The proposed approach is thus an important innovation for scientists working on strain characterization and substrate optimization as providing accurate forecasts will improve reproducibility and efficiency in early-stage bioprocess development.

## 1. Introduction

Early-stage bioprocess development refers to a phase in establishing a biotechnological production system that concerns optimizing strains and cultivation conditions. Shake flask experiments are simple, inexpensive, and easy to parallelize [1,2], and hence are an important aid for strain characterization and media optimization. Shake flasks come, however, with the drawback that measurements are mostly obtained offline by invasive sampling methods. Offline sampling can have side effects on cultivation conditions that are, for example, affected by a drop in dissolved O_2_ (DO) [3] or a shift in temperature. Experiments with novel strains or altered substrates are furthermore hard to judge in advance. The experimental outcome may thus be affected by a lack of important nutrients or critically low oxygen levels [1]. Missing the right time to harvest may further mislead the search for optimal strains and cultivation conditions [3,4]. The advent of innovative sensor technologies enabled in recent years optical, non-invasive methods to measure biomass (BM), O_2_, CO_2_, or pH. Shake flask experiments are thus increasingly monitored in an online fashion. Leaving cultures undisturbed increases product quality [3] and the reproducibility of process development [5,6,7,8].

In the pharmaceutical industry, Process Analytical Technology (PAT) and the Quality by Design (QbD) approach, proposed by the United States Food and Drug Administration (FDA), help to understand manufacturing processes scientifically. PAT and QbD are in addition required to assure the high-quality demands in pharmaceutical settings and may thus reduce rejects and reprocessing efforts [9,10,11,12,13]. Real-time monitoring of important process parameters is an important part of the respective FDA guidelines. Although the FDA recommends predicting process behavior, only a few publications, software solutions, or other methods attempt to forecast the dynamic evolution of bioprocesses over time [9,10]. There are several implementations of soft sensors, which predict unobserved process states like biomass from easily accessible input parameters [14,15]. Fermentation processes, which depend on the behavior of microorganisms and their interaction with the substrate [1], are often prone to variability. Despite being invaluable prerequisites for process optimization, only a few publications are devoted to forecasting of bioprocess signals [16,17,18]. Shake flask cultures deserve particular attention in this context. While online monitoring aids documentation of experiments and allows detecting unfavorable situations, insight into processes is only gained retrospectively. Considering measured online signals in an anticipatory context would certainly be desirable to detect suboptimal conditions before they happen. Knowing that undesired situations are on the horizon would allow scientists to react on time and either adjust the process parameters, or terminate the experiment. Knowing future BM levels and the optimal time to harvest in advance would furthermore increase yield, and improve product quality and the efficiency of process optimization.

**Figure 1:**
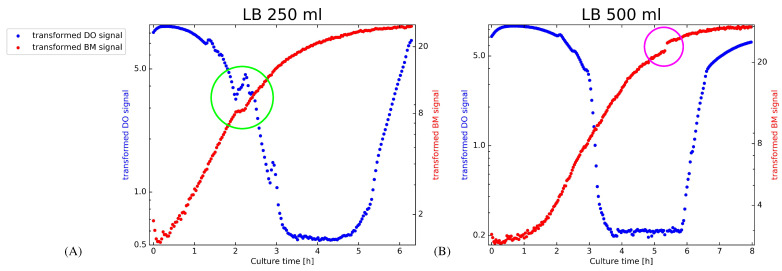
Visualization of transformed (see Section 2.3 for details) BM and DO online signals of *E. coli* cultivated in shake flasks. The left subplot (**A**) illustrates measurements obtained from an *E. coli* culture in 250 mL LB medium. An *E. coli* culture in 500 mL LB medium is visualized in subplot (B). Both cultures show indications of non-stationary growth. Subplot (A) uses a green circle to accentuate a period of reduced growth and increasing DO levels which in combination suggest that *E. coli* modifies its metabolism in response to a limitation of a nutrient [21]. In subplot (**B**), a magenta colored circle emphasizes a step in the BM signal, which is likely a technical issue. Both subplots show that shake flask cultures may modify their growth characteristics unpredictably.

A particular difficulty for in silico predictions of shake flask experiments is the inherent lack of control over the composition of the medium while cultivating biomass. We may thus find the composition of the substrate to change adversely and the cultivated cells to adjust their metabolism correspondingly [19,20]. Changes in metabolism will likely manifest in adapted growth dynamics and hence non-stationary behavior. Examples that illustrate non-stationary growth are shown in Figure 1. Subplot (A) accentuates a transient phase with a green circle where the growth of an *E. coli* culture slows down while the DO level increases at the same time. In combination, these patterns suggest that change is most likely caused by a limitation of an important nutrient and *E. coli* responds by adapting its metabolism. Subplot (B) in Figure 1 illustrates a step in the growth curve, which is most likely caused by a sudden change in the response of the optical sensor. The signals in Figure 1 provide evidence that predictive approaches should not rely on stationarity. If a combination of novel strain and substrate causes adverse growth conditions, predictive monitoring has to adapt swiftly and adjust the predicted BM and DO profiles.

Forecasting of time series can be approached by non-parametric or model-based methods. Non-parametric forecasting fits flexible models like neural networks to training data [22]. While non-parametric approaches allow representing a wide spectrum of nonlinear behavior [23], reliable parametrization requires large sample numbers and makes their application to early-stage bioprocess development challenging [17]. Non-parametric models are in addition restricted to interpolation tasks and cannot extrapolate. When investigating novel strains or substrate compositions, even models which represent past measurements well may thus fail in predicting how future BM or DO levels evolve.

Model-based forecasting relies on specific mathematical functions which employ a mechanistic understanding of temporal behavior. An important aspect of facilitating predictive monitoring in early stage bioprocess development is to obtain adequate representations of how biomass evolves. It is well known that the growth of many organisms can be described mathematically by the Monod equation, the Gompertz, or the Logistic function [24,25]. Gompertz or Logistic growth models are in comparison with non-parametric methods less flexible. The small number of parameters is however easier to adjust, the models come with an intrinsic interpretation and, in certain limits, with extrapolation capabilities. Parametric growth models are thus very appealing for predictive monitoring of shake flask cultures and can represent growth (e.g., for BM) and decay or reduction (e.g., for DO).

To parameterize growth models in non-stationary situations as illustrated in Figure 1, scientists proposed various methods that descend from the Kalman Filter (KF) [26]. While the original Kalman Filter is restricted to linear Gaussian settings, extended Kalman filters (EKF) and particle filters (PF) [27,28] can be applied to nonlinear and non-Gaussian problems and became therefore important for application in biotechnology [14,15]. In this paper, we apply a novel PF implementation that fits Gompertz or Logistic growth models in an online fashion to BM and DO signals, which are obtained while *E. coli* grows in shake flasks. The approach provides gradually improved predictions of future BM and DO signals of ongoing experiments. This is a substantial improvement over conventional post hoc parameterized static growth models, which provide only retrospective insight. The presented approach additionally allows for incrementally improved predictions of the timing of important events. Applying the PF to BM signals is useful to determine in advance when to stop the culture to harvest optimally. Applying the PF to DO signals provides similarly an advance notification at which time a critically low DO level will be reached. The proposed approach innovates current practice in early stage bioprocess development in two important aspects.

1. Unlike static models, which cannot be applied without preexisting data, the proposed PF workflow extends predictive monitoring to shake flask cultures, which assess novel strains and substrate compositions.
2. The ability of the PF to adapt swiftly to altered growth characteristics without user intervention allows predictive monitoring to cope with non-stationary situations, which are illustrated in Figure 1.

The materials and methods section and Chapter 2 in the supplement S1 contain all necessary details to reproduce our findings. Reproducibility is further aided by making the PF implementation for inferring Logistic and Gompertz models available in a GitHub repository under a GPL v3.0 license. The results section provides conclusive evidence that PF based predictive monitoring has great potential to improve strain and substrate optimization. Visualizations of PF internals corroborate the importance of considering automatically adapted state noise levels for all model parameters. The accuracy of predicted signals and event times demonstrates that PF based predictions quickly become more accurate than predictions of static models. Unlike the PF, static models have to be parameterized with data from a replicate culture that was obtained under the same conditions as the test culture. It is worth noting that static models fit under such conditions are a theoretical and indeed tough competitor as the pilot data which is used for model fitting is in practice unavailable when investigating novel strains. The reported evaluations suggest, therefore, that PF based growth model inference has great potential to improve early-stage bioprocess development where shake flasks are an important tool for research and development [1,29]. PF based predictive monitoring is in particular a viable extension in situations like [3], where data from shake flask experiments is already collected in an online fashion.

## 2. Materials and Methods

### 2.1. Shake Flask Experiments

Unbaffled single-use shake flasks with 250 and 500 mL volume (Corning Inc., New York, NY, USA) were placed on a shake flask reader (SFR) vario for obtaining back scattered light measurements (PreSens, Regensburg, Germany) and dissolved oxygen (DO). All cultivation experiments were performed with the *E. coli* clone W3110 (thyA36 supO λ−, ordering number at the German collection of microorganisms and cell cultures DSM: 5911). Two complex media were used for culturing these *E. coli* clones. The lysogeny broth (LB) medium without glucose consists according to Lennox of $5\;g\;L^{-1}$ yeast extract (Y1625, Sigma-Aldrich, St. Louis, MI, USA), $10\;g\;L^{-1}$ tryptone (95039, Sigma-Aldrich), and $5\;g\;L^{-1}$ sodium chloride (S9888, Sigma-Aldrich) [30]. All components were mixed in deionized H_2_O and sterilized by autoclaving. The terrific broth (TB) medium consists of $24\;g\;L^{-1}$ yeast extract (Y1625, Sigma-Aldrich), $20\;g\;L^{-1}$ tryptone (95039, Sigma-Aldrich and $4\;\mathrm{mL}L^{-1}$ glycerin (49770, Sigma-Aldrich), which were mixed in 900 mL deionized H_2_O [31]. A tenfold concentrated phosphate buffer, composed of $0.17\;\mathrm{mol}L^{-1}$ KH_2_PO_4_ (P5655, Sigma-Aldrich) and $0.72\;\mathrm{mol}L^{-1}$ K_2_HPO_4_ (P3786, Sigma-Aldrich), was mixed in 100 mL deionized H_2_O. Both solutions were autoclaved separately and added together after the solution cooled down below $60\;{}^{{}^{\circ}}C$. In order to quantify signal variation at the start of the experiments, the optical density at 600 nm (OD${}_{600}$) was determined in all experiments using a SmartSpec Plus photometer (Bio-Rad, Hercules, CA, USA). For all experiments in TB medium initial glycerol concentrations were measured with a Cedex Bio metabolite analyzer (Roche Diagnostics, Mannheim, DE, Germany). Cultures were collectively performed at $37\;{}^{{}^{\circ}}C$ and 50 mm shaker amplitude in a Multitron Pro shaking incubator (Infors HT, Bottmingen, CH, Switzerland). Variations in shaking rate, flask and filling volume, and medium and inoculum conditions are summarized in Table 1.

### 2.2. Sensor Technology

**Figure 2:**
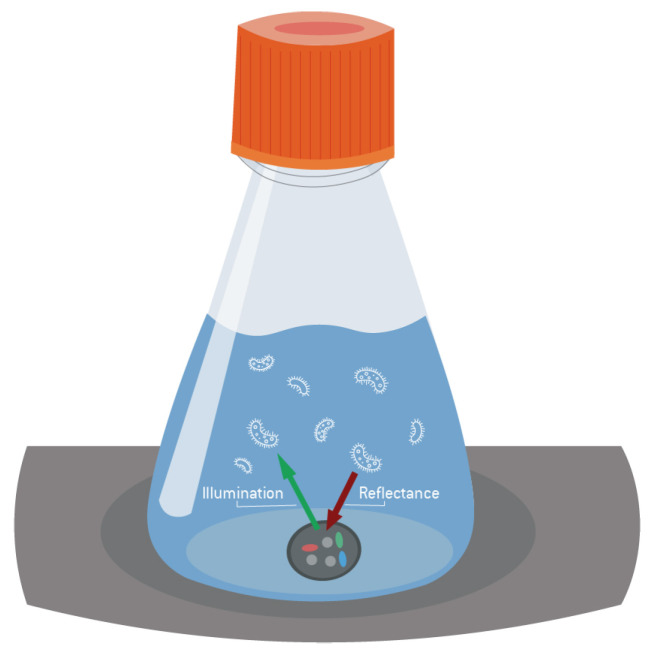
Illustration of the back scatter measurement principle of the SFR vario. The light emitted by LEDs is proportionally reflected by the cells or microorganisms in the suspension.

DO was measured using an oxygen-sensitive fluorescent membrane excited by modulated light in the kHz range of about 630 nm. As oxygen causes fluorescence quenching, a phase shift proportional to the oxygen partial pressure can be detected. For determining online measurements of BM concentrations we relied on scattered light measurements as are provided by the commercially available SFR vario (PreSens, Regensburg, Germany) device. The sensor module consists of a red LED (λ=630 nm) and a photo-diode at a nearly $180{}^{{}^{\circ}}$ angle. Further details on the sensor can be found elsewhere [32]. The optical system is positioned under the clamp of the shake flask at the edge of the curvature underneath the bottom of the flask. As is illustrated in Figure 2, the emitting light is transmitted through the transparent bottom of the shake flask and is reflected by particles resulting in a sensor signal being proportional to BM. The rotary shaking results in varying liquid heights over the sensor. Therefore, an acceleration sensor constantly measures the position of the whole system to determine the time and length of the measurement pulse [33]. Up to four devices were positioned in one shaker platform. Data transmission is achieved wireless using a Bluetooth standard.

### 2.3. Data

To assess PF inferred growth models for application in early stage bioprocess development, we use the experiments listed in Table 1. All cultures were done on *E. coli* clone W3310 in different volumes of LB and TB media. BM and DO signals were recorded with the sensors we describe in Section 2.2 in an online fashion. BM measurements are captured as Attenuation Unit (AU), which corresponds to a raw back scatter signal from the optical sensor. The dissolved oxygen (DO) signal is provided in percent of the maximum value. To avoid large values leading to numerical problems during model fitting, we divided the AU sensor signal by a constant factor of 1000. The DO signal was for the same reason divided by 10. Previous work, which is summarized by [25], considered Gompertz and Logistic growth models to represent biomass on a nominal and a logarithmic scale. Pilot evaluations on the LB 250 mL culture revealed that the log scale signals lead to improved Gompertz and Logistic model fits. We hence follow the majority of propositions in the review in [25] and consider in the remainder of this paper the rescaled BM and DO signals on a log scale.

### 2.4. Software

The commercially available software PAS-X Savvy 2021.03 (Werum IT Solutions-Division in Cyght, Vienna, Austria) and Python 3.79 (Python Software Foundation, Available online: https://www.python.org/, accessed on 31 July 2021) was used for data management, algorithm development and data analysis.

### 2.5. Models for Growth and Decline

In this work, the Gompertz and the Logistic function are considered as a mathematical basis for PF based predictive monitoring of BM and DO. Both functions are commonly used to model growth patterns of tumors, plants, or bacteria [34,35,36,37].

We parameterize the Gompertz function (Equation (1)), and the Logistic function (Equation (2)) according to the suggestions in [25], Table 2. The three parameters of the Gompertz

(1) y=k·exp(−exp(b−ct))

and the Logistic

(2) $$y=\frac{k}{1+\exp(b-ct)}$$

function can be directly identified from measurements. We use *k* to refer to the maximum value, *t* to denote process time and *b* and *c* to parameterize the transition dynamics.

At this point, it is worth noting that Gompertz and Logistic models allow the representation of monotonically increasing (b>0 and c>0) as well as monotonically decreasing functions (b<0 and c<0). Flipping the signs of *b* and *c* corresponds to mirroring both growth functions around a straight line which is parallel to the *y*-axis and passes through t=b. We may hence evaluate suitably parameterized Gompertz and Logistic functions for predictive monitoring of rising BM and declining DO levels.

### 2.6. Particle Filter Workflow

The well known Kalman filter (KF) [26] has a long tradition as a method to estimate the unknown state of time-varying linear dynamical systems. The interpretation of KFs in [38] as a recursive Bayesian inference method stimulated generic application to online parameter learning in non-stationary situations. To generalize beyond linear Gaussian settings, researchers have quickly developed parametric approximations like the extended [39,40], the unscented [41,42] and the variational [43,44] KF. Stimulated by increased computing power and advances in Monte Carlo integration, particle filters which combine importance sampling (e.g., [45], (p. 122)) with the ideas of Kalman filtering emerged in the 1990s as an even more general approach to online parameter inference [27,46,47,48,49].

**Figure 3:**
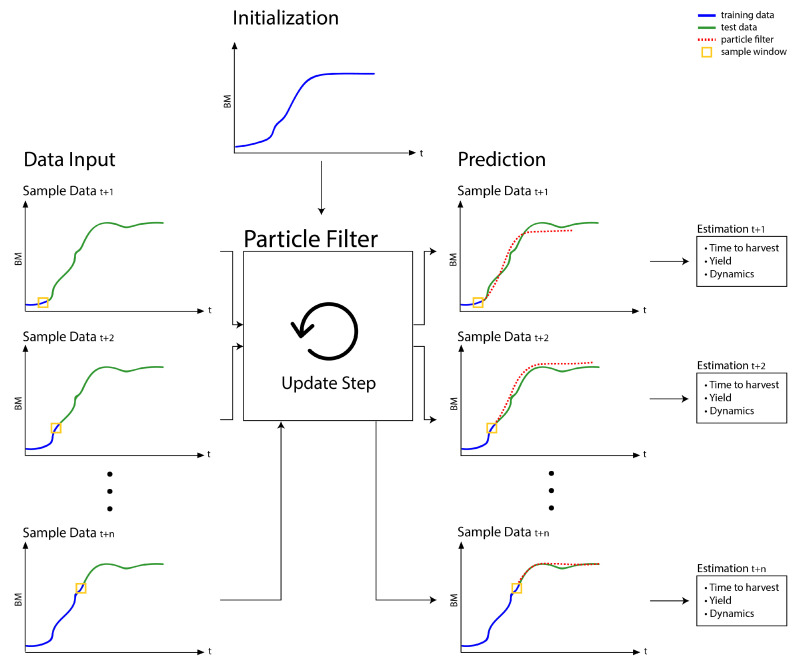
Workflow for predictive monitoring of BM in strain and substrate characterization experiments. The workflow consists of 4 steps: (1) initialization, (2) data input, (3) update step and (4) prediction. The initialization step is performed once before applying the PF to a monitoring task. The other steps are repeated as soon as new measurements arrive until the growth culture is terminated. Blue lines illustrate training data that is or was used for updating the PF. The green lines (test data) represent future data. The test data represent the yet unobserved samples of real life applications and constitute the ideal target values to be predicted by the PF. The PF predictions, which depend on initialization and all previous update steps, are illustrated as red dotted lines. A yellow rectangle illustrates a sliding sample window that contains the data which governs the PF update step. Besides predicting future values, the parameters of the Gompertz or Logistic growth models provide running estimates of temporal forecasts and growth characteristics.

An overview of the proposed PF workflow, which learns to predict BM is shown in Figure 3. Particle filtering (particle is a synonym for Monte Carlo sample) of the coefficients of Gompertz and Logistic growth functions starts by initializing the PF. To obtain unbiased performance evaluations for situations which scientists face in strand and substrate optimization, we use different cultures for PF initialization (Figure 3, subplot “Initialization”) and updating. To speed up convergence, initialization and PF updating should use reasonably similar growth profiles.

After initialization, the PF is applied in an online fashion to individual measurements as soon as they arrive. Individual PF updates are shown in Figure 3 as subplots “Data Input”. The blue lines illustrate past measurements that were previously used to update the PF (training data). The green line (test data) indicates future observations. In real life application, the actual values of all test data samples are unknown as they represent future BM (or DO) values. In the laboratory, newly measured samples will immediately be used for a PF update step before we predict estimates of the future growth curve. Since this paper wishes to evaluate the performance of the proposed PF workflow, the experiments are used retrospectively. Providing unbiased performance assessments of applications to strand and substrate optimization is warranted by excluding measurements from performance evaluation, once they were used for updating the PF.

The update step of the PF re-adjusts the growth model parameters to fit the data that is currently in the sample window (in Figure 3 illustrated as a yellow rectangle). As a result of performing update steps, the predictions (red dotted lines) become increasingly more accurate. The gradually improved performance is hinted in Figure 3 in the subplots “Prediction”. In contrast to a SM, which is by definition fit retrospectively to an entire growth profile, the PF can adjust to altered growth characteristics. Differences in growth characteristics are, for example, caused by modifying the substrate or exchanging the strain. Parameter adaptation is, however, also required, if a metabolic change results in a culture adapting its growth dynamics (e.g., the accentuated regions in Figure 1). Since data is not available before performing a culture, statically fit growth models (SM) can, unlike the proposed PF workflow, not be optimized to represent growth profiles of altered protocols. Statically fit growth models will therefore provide, in general, poor representations of new data. SMs can furthermore not react to changing dynamics and will inevitably fail to predict future values correctly. A PF based workflow should thus be better suited for predictive monitoring of biotechnological shake flask cultures. Predictive insight is, however, not restricted to future BM or DO values. The PF is equally suited to provide running estimates of optimal harvest times or critical event timing (e.g., critically low DO levels). We refer readers who are interested in implementation details of the PF workflow to the supplementary information Chapter 2 in the supplement S1, which accompanies this paper.

### 2.7. Initialization of the Particle Filter and Static Model Fitting

A quantitative evaluation of PFs for predictive monitoring of shake flask experiments which are typical for strain characterization and substrate optimization depends on a strong competitor. To obtain such competition, we fit a static Logistic or Gompertz growth model to a replicate culture, which was grown under identical conditions as the culture under test. To obtain realistic performance estimates of how the proposed PF copes with predictive monitoring of strain and substrate optimization, the PF should be initialized in an unbiased manner. All assessments therefore use cultures for PF initialization and filtering that were grown under different conditions. Static models have thus the advantage that predictions of test samples contain information from an entire growth profile, which was previously grown under identical conditions. Accuracies of SM based predictions therefore assess the reproducibility of growth profiles within protocols. As we evaluate PF based predictive monitoring of cultures which assess novel strains or substrate compositions, such training data is not available in real applications. The performance characteristics of SMs are thus idealistic and indeed a strong competition for PF based predictions.

Inferring static models and PF initialization apply a Markov Chain Monte Carlo (MCMC) method. MCMC considers the parameters of the chosen growth model (Gompertz or Logistic) as samples. The approach iterates over updates that guarantee that MCMC samples represent a distribution of growth model parameters which allow for accurate predictions of the growth profile that was used as training data. To simplify implementation, we use the previously discussed PF updates as a batch version of sequential importance resampling [46].

- The time of sample collection and the respective transformed BM or DO signal of a complete culture are randomly reshuffled and used as training data.
- After initializing the samples with randomly drawn Gompertz or Logistic model parameters, MCMC itself is just an application of the PF updates in Figure 3, however, on reshuffled growth profiles.
- By reshuffling the growth profiles, the sample window of the PF will always capture the dynamics of the entire growth profile. The generated samples are thus adapted to capture the dynamics of the entire profile and infer indeed a static model.

Similar to the PF, this procedure allows for quantitative predictions of BM or DO values or for event time predictions. While PF initialization and fitting SMs use the same algorithm, they differ substantially in which data is used for inference. Static models are used for predicting values of technical replicate cultures that were grown under identical conditions. The PF should however provide unbiased predictions of how a novel combination of strain and substrate grows. The PF therefore uses initialization and filtering data from different protocols.

### 2.8. Predictive Accuracy

To compare PF inferred with statically fit growth models in shake flask experiments we assess predictions with quantitative metrics. To obtain unbiased performance estimates only future values of BM and DO levels are considered. Having used samples 1 to *n* at time $t_{1}$ to $t_{n}$ and the corresponding signals $y(t_{1})$ to $y(t_{n})$ for filter updates, unbiased estimates of model performance can be obtained for all predictions at future time points $T=[t_{n+1},\ldots ,t_{N}]$. In line with the notation in Chapter 2 in the supplement S1, we use $\hat{y}\left(c_{\tau }\right)$ for $c_{\tau }\in T$ to denote the expectation of the predicted quantity at the future time point $c_{\tau }$. Denoting the corresponding measured value as $y(c_{\tau })$ the assumed Gaussian noise model suggests that we may evaluate predictions by a sum of squares metric, ssd as

$$ssd=\sum_{c_{\tau }\in T}{\left(y\left(c_{\tau }\right)-\hat{y}\left(c_{\tau }\right)\right)}^{2}.$$

Another metric of model performance is the mean squared error, mse

(3) $$mse=\frac{\sum_{c_{\tau }\in T}{\left(y\left(c_{\tau }\right)-\hat{y}\left(c_{\tau }\right)\right)}^{2}}{N-n},$$

which has the advantage of providing comparable values, while the particle filter progresses through the data and hence the metric which we use for evaluations.

### 2.9. Statistical Significance

To rule out that reported preferences are observed by chance, quantitative comparisons of predictive accuracy are assessed for statistical significance. Achieving optimal power in detecting true improvements by PF inferred growth models uses the fact that the predictions of PF and SM for the same $c_{\tau }$ are paired. To tie comparing mse values with a significance test, the squared residuals $\epsilon \left(c_{\tau }\right)={\left(y\left(c_{\tau }\right)-\hat{y}\left(c_{\tau }\right)\right)}^{2}$ from Equation (3) are considered as a test statistic. To simplify notation, we denote the PF based models with index PF and the SM fits with index SM. Both models lead hence for time $c_{\tau }$ to the squared residuals $\epsilon_{PF}\left(c_{\tau }\right)$ and $\epsilon_{SM}\left(c_{\tau }\right)$. After pairing, this provides us with $s\left(c_{\tau }\right)=\epsilon_{PF}\left(c_{\tau }\right)-\epsilon_{SM}\left(c_{\tau }\right)$ as per sample statistic. To avoid potentially unjustified distribution assumptions about $s(c_{\tau })$, significance considerations apply a non-parametric test. Since we are interested in quantifying superiority of PF based predictions we assess the null hypothesis that the expectation $E{\left[s\left(c_{\tau }\right)\right]}_{p(s\left(c_{\tau }\right)|D)}$ under the observed distribution is zero against the alternative hypothesis $E{\left[s\left(c_{\tau }\right)\right]}_{p(s\left(c_{\tau }\right)|D)}<0$. The *p*-values of this test are obtained in a non-parametric fashion by generating samples under the null hypothesis $p(s\left(c_{\tau }\right)|D_{0})$ by randomly exchanging $\epsilon_{PF}\left(c_{\tau }\right)$ and $\epsilon_{SM}\left(c_{\tau }\right)$ or alternatively the sign of $s(c_{\tau })$. Repeating the permutation *K* times to draw from the null hypothesis we count the number of instances, κ, where $E{\left[s\left(c_{\tau }\right)\right]}_{p(s\left(c_{\tau }\right)|D_{0})}<E{\left[s\left(c_{\tau }\right)\right]}_{p(s\left(c_{\tau }\right)|D)}$ and get p=κ/K as *p*-value that the improvement of PF based predictions can be explained by chance. To obtain accurately resolved *p*-values, we use $K=10^{5}$ permutations to determine κ. Multiple testing is considered by mapping all *p*-values to Benjamini Hochberg false discovery rates (FDRs) [50]. To claim significantly improved predictions for the particle filter inferred growth model, the FDR has to be smaller than 0.05.

### 2.10. Event Time Assessment

Predicted event times are compared with post hoc estimates, which represent the optimal target times. We define the time when the culture reaches 95% of the maximal BM value as the optimal time to harvest. We furthermore consider reaching 20% of the initial value of DO as critically low O_2_ concentration and compare the corresponding temporal predictions from SM and PF inference with respective post hoc estimates. To estimate optimal target event times, the BM and DO sensor values are first smoothed with a Savitzky Golay filter [51]. The filter is parameterized by setting the polynomial order to 6, the window length to 21 and uses mirroring mode for initial and terminal signal windows. After smoothing, a suitable value for the target time is estimated heuristically as the time when the smooth signal is closest to the above fractions of the signal maximum. PF predictions of event times are compared with post hoc estimates with errors in event time being quantified as difference

(4) $$td=t_{pred}-t_{opt},$$

where $t_{pred}$ refers to the SM and PF predicted time (see Chapter 2 in the supplement S1 for details) and $t_{opt}$ refers to the optimal target time, which we estimate from the smoothed signal. The implications of missing the optimal event time on yield and dissolved O_2_ levels are reported as relative differences between the smooth signal values at the predicted event time and the post hoc estimate, $\left(y\left[t_{pred}\right]-y\left[t_{opt}\right]\right)/y\left[t_{opt}\right]\;\cdot \;100\%$. Symbol *y* denotes here the smoothed time series after applying the Savitzky Golay filter.

### 2.11. Hyperparameter Tuning

Applying the proposed PF depends on specifying a few hyperparameters that have an influence on model performance. Model inference sets the hyperparameters of the Gamma densities in Figure S2 in the supplement S1 to g=0.1, h=1, α=0.1, β=1, a=0.1 and b=1. These parameter values are non-informative and hence universally applicable. Parameter δ in the proposal of the Metropolis Hastings update of γ (Equation (17) in the supplement S1) is set to 0.9. The two *E. coli* cultures, which were done in 250 mL LB medium, are used for calibration and initialization steps.

A first calibration step on the two LB 250 mL cultures in Table 1 found that the PF operates best when presenting the BM and O_2_ sensor signals on log scale. Considering log transformed BM agrees with previous suggestions that Logistic and Gompertz growth models are best suited when BM is mapped to a logarithmic scale [25]. Additional calibration steps were used to optimize the size of two sliding windows which are essential for operating the proposed PF. One window concerns the number of samples in a sliding window which defines the dimensions of $y_{t}$ and $\xi_{t}$ in Equation (9) in the supplement S1 and Equation (10) in the supplement S1. We refer to this window as sample window. The second window concerns the number of most recent particles, which we use for updating the state noise parameters ν, γ and Λ in Figure S2 in the supplement S1. We refer to the second window as the state noise window. We allowed both window sizes to vary among 5, 10 and 20. Assessments on the two LB 250 mL cultures yield for the data window a size of 20 and for the state noise window a size of 10 samples as the respective optima.

#### 2.11.1. Unbiased Evaluation

The validation experiments in the results section of this paper assess different aspects of PF inferred growth models. To provide unbiased results, initialization uses *E. coli* cultures which differ from the culture which we assess during predictive monitoring. Applying PFs for time series analysis uses past samples for parameter updates and predicts future behavior. To provide in this situation unbiased assessments we split the data into ten consecutive intervals. After using the data in an interval for updating the growth model parameters, we use the resulting particles to provide predictions for all future time points of the respective *E. coli* culture and use the time matched future observations of BM and DO for performance evaluation. To compare the PF to a different predictive approach, independent predictions on the same time points are also obtained with SMs. To provide unbiased results, a different *E. coli* culture that was grown under identical conditions (Table 1) is used to parameterize the SM. PF based inference can in principle be started with particles that are randomly drawn from any valid distribution. To speed up tracking, it is however beneficial to initialize with a particle distribution which is not too far off the (unknown) target distribution. To properly assess the PF when applied to predictive monitoring of substrate and strain optimization, we use both LB 250 mL cultures for initialization and exclude this data from performance evaluation. Table 2 lists how we combine the data of different growth cultures to obtain unbiased evaluations. PF performance in predictive monitoring of novel experiments is hence assessed on two cultures with *E. coli* grown in 500 mL LB medium and on three cultures where we use 500 mL of TB medium. As we consider all individual measurements of the growth profile for performance evaluation, five replicate simulations are sufficient to conclude how PF based predictive monitoring copes with strain and substrate optimization.

#### 2.11.2. Validation Experiments

The validation experiments in the results section assess different aspects of PF inferred growth models. Accuracies of BM predictions are quantified after having used 30%, 40%, …up to 80% of the time series for growth model inference. The remaining future observations allow us to provide unbiased performance estimates. The assessment of the DO time course follows a similar strategy. Evaluation is, however, only visualized for predictions that use between 30% and 60% of the data for inference. We decided to stop at 60%, as there is little value in learning to predict zero levels, which we observe for the final 35% of the DO time course (see Figure 1).

1. To demonstrate the importance of adapting the state noise levels in the proposed PF implementation, we assess the filter internals while adapting to the transformed BM signal of the LB-500 mL-2 experiment (Figure 1, subplot (B)). The depicted results are obtained after initializing the PF on the LB-250ml-2 experiment before we switch to the LB-500 mL-2 experiment. The implications of estimating appropriate state noise levels are best captured in a synchronized view of how the particle filter operates. We illustrate to this end how the state noise levels of the filter, the transformed BM signal and a windowed mse evolve. The mse values are in this case estimated from one step ahead predictions with a sliding window of 15 samples.
2. Evaluating PF inferred growth models for predictive monitoring during strain and substrate optimization has to mimic situations where little is known about how the culture evolves. To obtain unbiased assessments for such use cases, we use both LB 250 mL cultures for PF initialization and switch the PF for evaluation purposes to the *E. coli* cultures that were grown in 500 mL of LB and TB medium (Table 2). Strain and substrate optimization implies that data of a culture that was obtained under respective conditions for fitting SMs is not yet available. Such situations hence prevent us in practice from using statically fit models for predictive monitoring. For evaluation purposes, it is however still informative to see how well PF based inference competes with SMs.

### 2.12. Derivation, Code and Data Availability

Implementation details of the particle filter for inferring Gompertz and Logistic growth models is available as supplementary methods File S1. The PF implementation, BM values, DO measurements and a jupyter notebook that illustrates the application of the PF to growth predictions is available on the GitHub repository https://github.com/psykacek/pf4grwth, (released on 31 July 2021) under a GPL v3.0 license. All data to reproduce our results are in addition available alongside with the paper as supplementary material File S2.

## 3. Results

The objective of predictive monitoring of shake flask experiments is providing accurate forecasts of how the combination of microorganism, strain and substrate affects growth. Scientific workflows in biotechnology would in addition benefit from advanced alerts of critical events, like when to harvest in an optimal manner. An inherent difficulty for implementing predictive monitoring of biotechnological shake flask experiments is demonstrated in Figure 1: suboptimal substrate composition and technical issues may cause growth profiles to violate stationarity. By noticing that change happens abruptly and is in general time dependent, we proposed to rely on PF inferred growth models for predictive monitoring. The objective of the results section is to provide experimental evidence that supports the conclusion that PF inferred growth models provide predictions that can improve shake flask workflows in biotechnology. We start by investigating the internals of the PF when inferring growth models from the BM signal values which we visualize in Figure 1 subplot (B). This investigation reveals that the adaptive state noise levels behave as expected and allow that the PF responds quickly to changing growth profiles. A subsequent investigation of PF based predictions of future BM values and DO provides insight into how PF based predictive monitoring can aid unforeseen or novel situations that occur during strain and substrate optimization.

### 3.1. Tracking Performance

**Figure 4:**
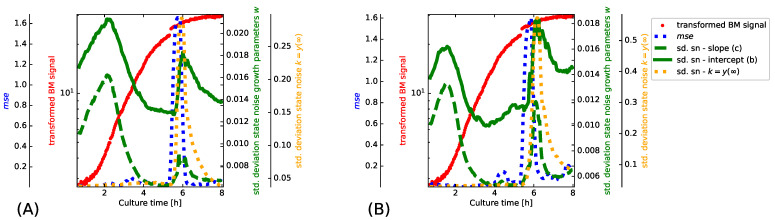
Tracking of a non-stationary transformed BM signal (see Section 2.3 for further details on the transformation). To gain insight into how the PF copes with non-stationary events, we illustrate the internals of the PF for a Logistic growth model in subplot (**A**) and for a Gompertz growth model in subplot (**B**). Superimposing the rescaled traces of BM, mse and the scale of the state noise of all growth model parameters shows how the PF alters in a situation dependent manner between stationary precision and efficient tracking. We plot the BM values as red dots, the windowed mse estimates of one step ahead predictions as blue dotted line, the standard deviations of the state noise for intercept, *b*, and slope, *c*, as solid and dashed green lines. and for the limit population size, *k*, as orange doted line. The role of the parameters *b*, *c* and *k* is apparent from Equations (1) and (2). We see in the graphs that the step in the BM signal after around 5 h cultivation time causes a substantial increase in mse. The PF responds to the resulting mismatch of the growth model by automatically increasing the standard deviations of all state noise distributions. Increased state noise levels allow the PF to “forget” past parameter values and to adapt quickly to the new situation. Observing the largest relative increase in state noise level for the limit population size *k* agrees with our intuition that a step in the transformed BM signal is best compensated by increasing this model parameter.

An essential aspect of the proposed PF is its ability to automatically adapt in a situation dependent manner between precise convergence and efficient tracking. This flexibility facilitates that the PF reacts efficiently to a change in growth dynamics. The proposed hierarchical model has the additional advantage that every model coefficient has its own state noise level. Inferring state noise levels furthermore avoids that we have to run time consuming validation tests to set these parameters to appropriate values. The directed acyclic graph in Figure S2 in the supplement S1 illustrates the relation between the growth model parameters and the state noise levels. By assuming the dynamics of transients being constant within a sliding window, the precisions (inverse variances) of the state noise distributions Λ, ν and γ may be inferred according to the propositions in Chapter 2.1.3 in the supplement S1. If growth is stationary, convergence to precise parameter distributions is facilitated by large Λ, ν and γ values. Such a scenario manifests in Figure 4 as small standard deviation of the state noise distributions. If tracking of non-stationary growth dynamics is required, the PF will automatically reduce Λ, ν and γ. The plots in Figure 4 illustrate such a transient phase in the LB-500-2 experiment shortly after cultivating for 5 h.

To visualize the internals of the PF while sequentially inferring how biomass evolves, we superimpose the BM values (red dots), windowed mse estimates (blue dotted line), the standard deviations of the state noise distributions for the parameters, *c*, *b* (dashed and solid green line) and the limit population size *k* (dotted orange line). The varying state noise levels which we observe in Figure 4 for the different model coefficients is a manifestation of unequal parameter scales [52,53]. Although a comparison of the two subplots in Figure 4 reveals a dependency of the state noise levels on the growth model, the overall trend is strikingly similar. The initial increase in state noise level for the parameters *b* and *c* suggests that the differences between LB-250-2 and LB-500-2 mostly manifest as altered growth dynamics. The decreasing state noise levels which we observe after around two hours suggest that growth of *E. coli* is stationary and the PF infers precise parameter distributions. After around 5 h into the experiment, the transformed BM signal experiences a step wise transient and the PF reacts by increasing all state noise levels. Increased state noise levels allow that the PF “forgets” past parameter values and adapts quickly to the new situation. Observing the largest relative increase for the state noise level of the limit population size *k* agrees with our intuition that a step in the BM signal is best compensated by increasing this model parameter.

### 3.2. Predictions and Accuracy

To gain insight into how PF inferred growth models innovate shake flask experiments in biotechnology, this section provides selected visualizations and a thorough evaluation of performance characteristics. Most shake flask experiments are in biotechnology used for strain characterization and optimization of growth conditions. Strain, substrate and parameters like temperature, shaker agitation rate and orbit affect growth dynamics and yield. Past experiments thus provide only a little information about what to expect in exploratory assessments of novel conditions. Statically fit models are adapted to predict growth profiles under specific conditions and therefore in general poor when predicting how biomass or dissolved oxygen evolve under modified experimental settings. To allow for predictive monitoring of shake flask experiments under modified conditions, the PF based predictor is adapted while the culture grows. Since online parameter estimation can always be applied, the results which we observe on a selected number of growth cultures are therefore representative for other experiments. In the language of data science, the evaluations which we report here assess generalization performance. As long as Gompertz and Logistic growth models are adequate representations of biomass, we may hence expect to obtain similar results.

**Figure 5:**
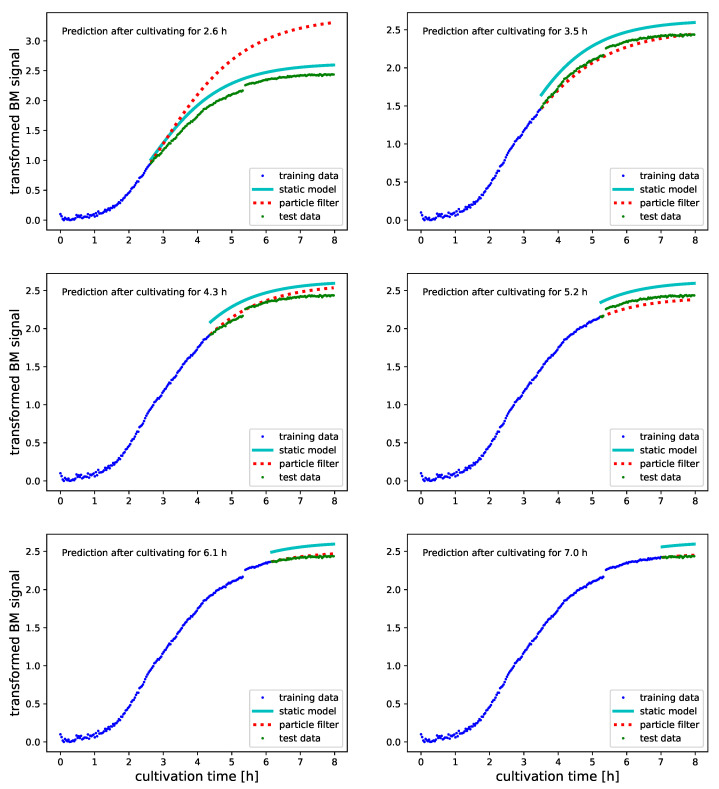
Predicted transformed BM values (see Section 2.3 for further transformation details) with Gompertz growth models for a culture in 500 mL of LB medium. Predictions are done after having used 30%, 40%, …and 80% of the signals for PF updating. All data which was used for updating the PF before obtaining the predictions is illustrated as blue dots. Green dots illustrate the true future BM values. Predictions which were obtained with a static Gompertz growth model that was parameterized on a replicated LB-500 mL culture are illustrated as cyan line. The predictions by the PF inferred Gompertz model are illustrated as dotted red line. Despite initializing the PF on a culture that was grown under different conditions the PF starts to outperform the SM after having used half of the data for parameter inference. A noteworthy observation concerns the step in the in BM signal which happens after around 5.5 h. While the step causes the PF predictions provided after 5.2 h to be off by a constant margin, the predictions provided after 6.1 h are fully recovered. The increase in state noise level which can be seen in Figure 4 after around 5.5 h allows the model to adapt quickly to the shifted curve.

**Figure 6:**
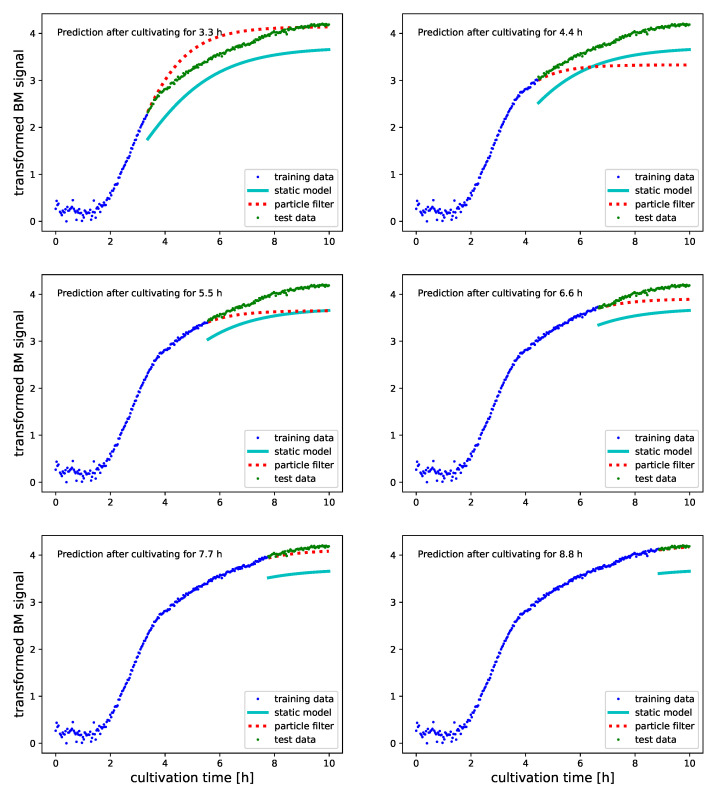
Predicted transformed BM values (see Section 2.3 for further transformation details) with Gompertz growth models for a culture in 500 mL of TB medium. Predictions are done after having used 30%, 40%, …and 80% of the signals for PF updating. All data which was used for updating the PF before obtaining the predictions is illustrated as blue dots. Green dots illustrate the true future BM values. Predictions which were obtained with a static Gompertz growth model that was parameterized on a replicated TB-500 mL culture are illustrated as cyan line. The predictions by the PF inferred Gompertz model are illustrated as dotted red line. The culture in 500 mL of TB medium confirms that even when initialized on a culture that was obtained under different conditions, PF based inference outperforms a statically fit growth model after having used 50% of the culture for adjusting the model. A second observation on TB medium concerns the reduction in growth dynamics which we observe in the BM signal around 4 hours after starting the experiment. Such transitions can only be captured by time varying growth model parameters. A SM which relies on a fixed stet of parameters will therefore provide a poor representation of the entire curve. Only by tracking change do we have a chance to provide at some point an accurate representation of the remaining growth phase.

**Figure 7:**
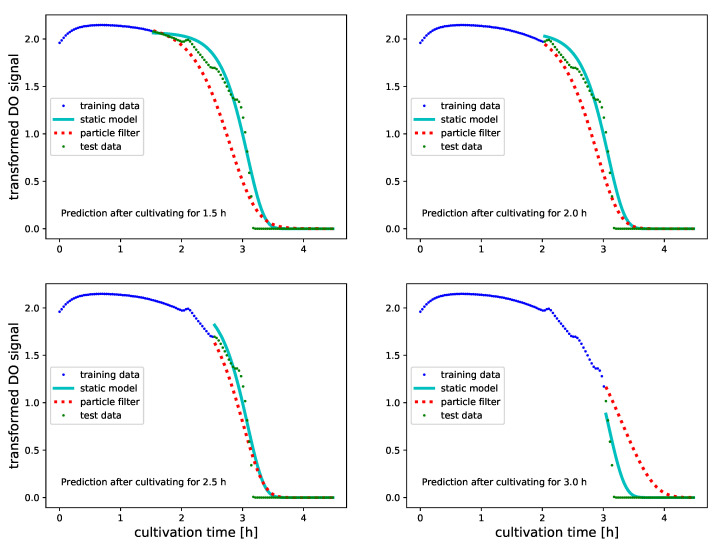
Predicted transformed DO values (see Section 2.3 for further transformation details) with Gompertz growth models for a culture in 500 mL of LB medium. Predictions are done after having used 30%, 40%, 50% and 60% of the signals for PF updating. All data which were used for updating the PF before obtaining the predictions are illustrated as blue dots. Green dots illustrate the true future O_2_ values. Predictions which were obtained with a static Gompertz growth model that was parameterized on a replicated LB-500 mL culture are illustrated as cyan line. The predictions by the PF inferred Gompertz model are illustrated as dotted red line. Although the PF based predictions of future O_2_ signals are not better than the predictions with static models, we obtain results which are in general comparable. The application of PF based growth models for predicting future O_2_ levels is therefore justified, if lack of data prevents fitting SMs.

**Figure 8:**
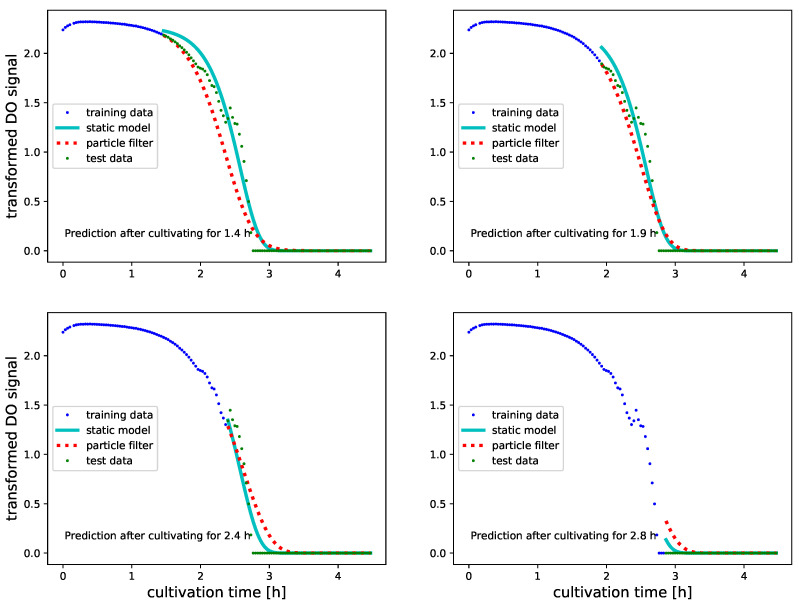
Predicted transformed DO values (see Section 2.3 for further transformation details) with Gompertz growth models for a culture in 500 mL of TB medium. Predictions are done after having used 30%, 40%, 50% and 60% of the signals for PF updating. All data which was used for updating the PF before obtaining the predictions is illustrated as blue dots. Green dots illustrate the true future O_2_ values. Predictions which were obtained with a static Gompertz growth model that was parameterized on a replicated TB-500 mL culture are illustrated as cyan line. The predictions by the PF inferred Gompertz model are illustrated as dotted red line. Although the PF based predictions of future O_2_ signals are not better than the predictions with static models, we obtain results which are in general comparable. The application of PF based growth models for predicting future O_2_ levels is thus justified, if lack of data prevents fitting static models.

To judge how PF inferred growth models perform in predictive monitoring, we visualize predicted BM and DO levels for selected experiments. The proposed particle filter infers Logistic and Gompertz growth models in an online fashion. Signal values and process time are therefore used for PF updates as soon as they are measured. Our expectation that predictions are continuously improved suggests investigating model predictions while time progresses. Figure 5, Figure 6, Figure 7 and Figure 8 illustrate to this end the predictions that arise after incremental updates of the model parameters. Predictions of future BM values are provided after allowing the PF to adjust on the first 30%, 40%, etc., up to 80% of the measured signal.

Predictions of future O_2_ levels are provided in a similar manner, however, after using the initial 30% to 60% of the measured signal for PF inference. Refraining from updating the PF further is motivated by observing that the O_2_ signals are approximately zero during the last 35% of the time course. Continuing to update the O_2_ predictor leads therefore to very accurate level zero forecasts and is of little interest for assessing the PF. An overall performance assessment of predictive monitoring of BM and DO signals is summarized in Table 3 and Table 4.

By using cultures for PF initialization and predictive monitoring that were grown under different conditions, we obtain insight how PFs cope with experiments that aim at strain and substrate optimization. The progress of predictive monitoring of novel shake flask experiments for the BM signal is shown in Figure 5 and Figure 6. The deviations between SM predictions and true BM values are a result of using different replicate cultures for model parametrization and testing and allow us to relate PF performance to experimental reproducibility. For both experiments, we initially obtain PF predictions that are outperformed by the SM. This is no surprise, as the SM was parameterized on an entire growth profile to predict the BM values of an experiment that was cultured under identical conditions (see Table 2 for how we pair training and test data). However, after using half of the culture for model inference, the PF consistently outperforms the SM and provides significantly improved predictions (see Table 3, columns log(MSE${}_{\mathrm{PF}})$, log(MSE${}_{\mathrm{SM}})$ and % sig). Another advantage of PF over SM based predictions can be seen in Figure 5 in the subplots which predict future BM levels at 5.2 and 6.1 h into the experiment. The prediction at 5.2 h into the experiment happens just before the step in the BM signal in Figure 1 subplot B. Except for a constant deviation after the step, the PF predicted BM levels are an excellent representation of the true growth profile. As the step in the signal happens unexpectedly, the prediction errors for later time points cannot be avoided. The real power of the PF is to cope with such events rather efficiently. As is shown in Figure 4, the proposed PF implementation increases the standard deviation of the state noise in response to the mismatch automatically and quickly adapts the growth model parameters to the new situation. The visualizations in Figure 5 show that already after 6.1 h into the experiment the PF managed to compensate for the modified growth characteristics and predicts future values accurately.

Figure 7 and Figure 8 illustrate predictive monitoring of DO signals of two different experiments. As is evident from Table 4, these illustrations are representative for the performance of PF on DO signals: unlike for BM where the PF outperforms the SM after a sufficient number of updates, the PF can not match the predictive accuracy of the static model. The improved predictions of SM suggest that DO profiles vary less than BM profiles when replicating experiments. Despite that on DO signals we can not improve the predictive accuracy of SM, the PF achieves almost the same level of accuracy. If applied to strain and substrate optimization data to fit SMs is not available. Obtaining similar performance levels with the PF is therefore good news as it shows that we may use PF inferred growth models for predictive monitoring of DO signals.

### 3.3. Event Notification

Predicting BM and DO levels provides quantitative guidance how *E. coli* cultures evolve. Notifications and advanced alarms of critical events are, however, even more important to improve product quality and reproducibility of shake flask experiments [1]. To investigate whether PF inferred growth models provide accurate event time predictions, we look at predictions of (1) the optimal time to harvest and (2) the time when DO levels in shake flasks reach a specified (low) level. Harvesting in an optimal and consistent manner is of great importance. Relying on reproducibly predicted harvest times allows us to decide in a principled manner about optimal strains and growth conditions. Predicting the time when critically low oxygen levels arrive is important to avoid or at least detect limitation of O_2_ which might reduce growth or cause acidic conditions [1].

The result of the metrics for event time assessment which were proposed in Section 2.10 are summarized for the PF and the SM as td${}_{\mathrm{PF}}$ [h], td${}_{\mathrm{SM}}$ [h], loss${}_{\mathrm{PF}}$ [%] and loss${}_{\mathrm{SM}}$ [%]. Table 3 shows the results for BM data and Table 4 on transformed DO measurements. Columns td${}_{\mathrm{PF}}$ [h] and td${}_{\mathrm{SM}}$ [h] report average differences between predicted event times and the post hoc estimates in hours. Implications of missing the theoretical optimum are summarized in columns loss${}_{\mathrm{PF}}$ [%] and loss${}_{\mathrm{SM}}$ [%]. The latter two columns report the average relative differences in the respective signal which is caused by the growth model prediction missing the optimal event time.

Verdicts about PF based growth models for predicting harvest time may be drawn from Table 3. When looking at the numbers in columns td${}_{\mathrm{PF}}$ [h] and td${}_{\mathrm{SM}}$ [h], it is evident that in comparison with the Logistic function, the Gompertz growth model provides comparable or more accurate harvest time predictions. This assessment holds irrespective whether we apply the SM or the PF for parameter inference. The accuracy of harvest time predictions depends in addition on the medium. This finding suggests that certain growth conditions like, for example, the TB medium, result in profiles which are less suited to be represented as *one static* Gompertz or Logistic function. Using PFs for inference will in such situations provide regionally adapted approximations, which will eventually converge to a final representation which captures the entire future growth profile adequately. The harvest time predictions of the PF *must* therefore at some point improve over the statically fit model. For the harvest time predictions in this assessment we find that the PF outperforms the SM after having used 40% of the LB-500 mL culture and 70% of the TB-500 mL culture for growth model inference. Once the PF improves over the SM, the average difference between predicted and post hoc estimated harvest time is less than 30 min. After completing 40% of the LB-500 mL culture, we provide a precise notification of “time to harvest” around five hours before the event happens. Accurate notifications about when to harvest can for the TB-500 mL culture be provided around three and a half hours in advance. Such advance notification time should in practice suffice to improve laboratory efficiency and the reproducibility of strain and substrate optimization.

A evaluation of PF and SM based event time predictions for DO levels is shown in Table 4. The numbers in columns td${}_{\mathrm{PF}}$ [h], td${}_{\mathrm{SM}}$ [h] show that critical event predictions with the PF are slightly worse than SM based predictions. This observation suggests that the DO profiles obtained for a replicate culture under identical conditions are more reproducible than the respective BM profiles. Noticing that the average differences between PF provided critical event time for DO and the post hoc estimate are with two exceptions less than 30 min suggests that PF based predictions are accurate enough to be useful. Shake flask experiments which target strain characterization or substrate optimization will thus also benefit from PF based predictive monitoring of DO levels.

## 4. Discussion and Conclusions

This paper started by hypothesizing that PF inferred Gompertz and Logistic growth models are optimally suited for predicting how BM and DO evolve in *E. coli* shake flask experiments which target strain characterization or substrate optimization. Established knowledge [19,20] and our own online measurements of BM and DO in *E. coli* shake flask cultures suggest that bacterial growth dynamics are subject to transient modification. Encouraged by similar applications in biotechnology [14,15], we propose PFs as an inference method of Gompertz and Logistic growth model parameters (the particles) to describe how the transformed BM and DO signals evolve. Figure 1 suggests that inference of growth models for BM and DO values must consider that the *dynamics* how model parameters change is subject to modification as well. The standard deviation of the state noise, which allows that PFs track non-stationary behavior should therefore be adapted while filtering progresses. To provide optimal forecasts of BM and DO for shake flask experiments, we hence propose a novel PF implementation for Logistic and Gompertz growth model inference. To cope with changing degrees of non-stationarity inference alongside the graph structure in Figure S2 in the supplement S1 extends conventional PFs in a hierarchical manner. To our best knowledge, this PF implementation for tracking the parameters of Logistic and Gompertz growth curves is new and in this paper for the first time evaluated for PF based predictive modeling of *E. coli* shake flask experiments.

PF inferred growth models are evaluated by predicting BM and DO levels of several *E. coli* cultures. An essential aspect of our evaluation is addressing the most important use case of shake flasks in biotechnology: strain characterization and substrate optimization. A preparatory investigation on independent data suggested that BM and DO are best represented on a log scale. To demonstrate that biotechnological shake flask experiments benefit from the proposed architecture, the internals of the PF and windowed mse estimates were monitored while fitting Logistic and Gompertz models to an *E. coli* culture, which shows a sudden step in the transformed BM values (cf. Figure 1 subplot (B)). Synchronized traces of BM measurements, mse values and the standard deviations of parameter specific state noise distributions illustrate how inference copes with non-stationary situations.

We observe in Figure 4 that the standard deviations of the state noise distributions increase quickly in response to a step in the transformed BM signal. Increasing state noise allows that the PF switches quickly from a stationary accuracy mode to efficient tracking. Observing the largest relative increment in the state noise level of the limit population size parameter agrees with the intuition that a step in the signal is best compensated by adjusting this model parameter. The results in Figure 4 lead to the conclusion that the proposed PF architecture is well suited for predictive monitoring of shake flask experiments.

- Different scales of growth model parameters are explicitly considered and allow for a parameter specific adaptation between convergence and tracking.
- Some phases of microbial growth follow static patterns and allow precise parameter inference. At other times, randomly occurring factors cause the growth dynamics to change considerably. Considering a window for estimating state noise levels adapts inference automatically to different transient dynamics. Predictive monitoring thus reacts efficiently to changing regimes.
- By adapting the state noise levels automatically, tedious validation experiments for calibrating the state noise levels are avoided and the proposed PF can be applied immediately.

To evaluate predictive monitoring quantitatively, Section 3.2 relies on mse and *p*-values which evaluate PFs against SMs for significantly improved predictive accuracy. To judge predictive monitoring under realistic conditions, we update the PF on every tenth of the time series and assess accuracy on all remaining observations of the culture. The performance metrics which we show in Table 3 for several *E. coli* cultures show that predictive accuracy improves continuously while filtering progresses. The second half of the growth process is of particular interest. We observe there that the PF inferred models significantly outperform SMs on all tested shake flask experiments. Although predicting DO does not provide the same level of improvement, we can still justify using PF inferred growth models because prediction errors are in general small.

An assessment whether PFs are useful for predictive monitoring of shake flask cultures should also investigate how accurately we predict the optimal time to harvest and the arrival of critically low O_2_ levels. While SMs misjudge harvest time by a constant interval, the same analysis of PF inferred growth models finds that harvest time predictions will eventually differ from post hoc estimates by less than 30 min. The accuracy of PF based harvest time prediction is sufficient to obtain yields which are only a few percentage off the optimal target. A similar investigation which predicts the time, when critically low DO levels arrive, shows no advantage for PF based inference. Despite that PFs cannot match the accuracy of SMs, the PF predicted arrival times of critically low DO levels are with two exceptions less than 20 min off target. PF based predictive monitoring of DO levels is hence helpful if shake flask experiments are used for strain and substrate optimization where experimental conditions are constantly changed and data for fitting SMs is in general not available.

By evaluating a novel approach for predictive monitoring of shake flask experiments on several *E. coli* cultures we demonstrate that particle filters allow to extend predictive monitoring to early stage bioprocess development. Using online measurements in an anticipatory context provides experts with additional guidance how to proceed when optimizing strains and substrates. Reliable quantitative predictions allow, for example, to terminate cultures early if we find that predicted BM values are suboptimal. Having means at hand which estimate the optimal time to harvest reliably improves yield and product quality. Exact timing allows us to schedule experiments in an optimal manner and has moreover the advantage of harvesting in a reproducible manner. Advanced notification about undesired situations like low DO levels allows scientists to react in a timely manner. The experimenter can either adjust the process parameters, or terminate the experiment.

PF based predictive monitoring of growth cultures has thus great potential to extend PAT and QbD to early-stage bioprocess development. The proposed methodology is in particular a viable extension if data from shake flask experiments is already collected in an online fashion. Providing code and a demo application under a GPL v3.0 license allows that scientists who work in bioprocess engineering explore PF based modeling of their own shake flask experiments. Although the presented analysis focused on *E. coli* the demonstrated ability to generalize between different growth conditions is not far from generalizing to other microorganisms. The main limitation of the current implementation is that growth should approximately follow the patterns of Logistic or Gompertz functions. Due to the flexibility of particle filters the proposed approach is, however, readily applied to fermentation processes which require other parametric models.

## Figures and Tables

**Table 1:** Overview of conducted experiments with *E. coli* clone W3310 in LB and TB media. All experiments were carried out at $37\;{}^{{}^{\circ}}C$ in a Multitron Pro (Infors HT, Bottmingen, CH) with a shaking amplitude of 50 mm. Values for OD${}_{600}$ and glycerin (Gly) are starting values.

Name	Media	Flask Size [mL]	Filling Volume [mL]	Shaking Rate [rpm]	OD_600_	Gly [g L${}^{-1}$]
LB-250 mL-1	LB	250	40	180	0.109	-
LB-250 mL-2	LB	250	40	180	0.109	-
LB-500 mL-1	LB	500	80	200	0.128	-
LB-500 mL-2	LB	500	80	200	0.130	-
TB-500 mL-3	TB	500	50	200	0.074	5.43
TB-500 mL-4	TB	500	50	200	0.076	5.45
TB-500 mL-5	TB	500	80	200	0.122	5.21
TB-500 mL-6	TB	500	80	200	0.116	5.26

**Table 2:** Overview of evaluation experiments.

SM Training	PF Initialization	SM & PF Testing
LB-500 mL-1	LB-250 mL-1 & 2	LB-500 mL-2
LB-500 mL-2	LB-250 mL-1 & 2	LB-500 mL-1
TB-500 mL-3	LB-250 mL-1 & 2	TB-500 mL-4
TB-500 mL-4	LB-250 mL-1 & 2	TB-500 mL-5
TB-500 mL-5	LB-250 mL-1 & 2	TB-500 mL-6

**Table 3:** Performance assessments of predictive monitoring of BM values.

Time [%]	log(MSE${}_{\mathrm{PF}})$	log(MSE${}_{\mathrm{SM}})$	% sig	td${}_{\mathrm{PF}}$ [h]	td${}_{\mathrm{SM}}$ [h]	Loss${}_{\mathrm{PF}}$ [%]	Loss${}_{\mathrm{SM}}$ [%]
Gompertz growth model on LB-500 mL							
30	−1.99	−3.83	0.00	0.57	0.48	2.41	2.17
40	−3.05	−3.77	25.00	−0.25	0.48	−1.79	2.17
50	−5.58	−3.87	100.00	0.40	0.48	1.92	2.17
60	−6.90	−4.10	100.00	0.17	0.48	0.84	2.17
70	−9.13	−4.19	100.00	0.26	0.48	1.30	2.17
80	−9.26	−4.26	100.00	0.20	0.48	0.98	2.17
Gompertz growth model on TB-500 mL							
30	−2.01	−0.87	83.33	−1.37	−0.55	−10.15	−7.75
40	−1.28	−1.05	66.67	−2.43	−0.55	−17.40	−7.75
50	−1.69	−1.30	33.33	−2.01	−0.55	−14.06	−7.75
60	−2.78	−1.59	100.00	−1.11	−0.55	−8.43	−7.75
70	−4.42	−1.88	100.00	−0.37	−0.55	−3.17	−7.75
80	−6.34	−2.04	100.00	−0.34	−0.55	−4.04	−7.75
Logistic growth model on LB-500 mL							
30	0.24	−3.74	0.00	−0.77	−0.40	−9.70	−4.28
40	−0.95	−3.74	0.00	−0.74	−0.40	−12.10	−4.28
50	−4.69	−3.88	50.00	−0.69	−0.40	−6.35	−4.28
60	−4.16	−4.24	50.00	−0.69	−0.40	−6.36	−4.28
70	−7.33	−4.46	100.00	−0.39	−0.40	−2.75	−4.28
80	−9.06	−4.56	100.00	−0.37	−0.40	−3.47	−4.28
Logistic growth model on TB-500 mL							
30	−0.96	−0.81	33.33	−3.23	−1.41	−25.13	−11.43
40	−0.67	−0.99	0.00	−3.34	−1.41	−25.49	−11.43
50	−1.31	−1.24	33.33	−2.57	−1.41	−18.86	−11.43
60	−2.32	−1.51	83.33	−1.59	−1.41	−11.42	−11.43
70	−4.05	−1.79	100.00	−0.64	−1.41	−5.03	−11.43
80	−5.98	−1.94	100.00	−0.13	−1.41	−1.75	−11.43

Column time [%] denotes the time which splits training and test samples in percent of the duration of the entire growth experiment. Columns log(MSE${}_{\mathrm{PF}})$ and log(MSE${}_{\mathrm{SM}})$ denote the average logarithm of the mean square prediction error for the particle filter (index PF) and for the statically fit model (index SM). The use of logarithms improves resolution and implies that smaller values correspond to better performance. Column % sig summarizes the *p*-value calculations in Section 2.9 by reporting the fraction of cultures for which the PF leads to a significant improvement over the SM. Columns td${}_{\mathrm{PF}}$ [h] and td${}_{\mathrm{SM}}$ [h] are calculated according to Equation (4) and report the differences between predicted harvest time and the post hoc identified value in hours. The implications of differences between harvesting as predicted and the theoretical optimum are summarized in columns loss${}_{\mathrm{PF}}$ [%] and loss${}_{\mathrm{SM}}$ [%]. Averaging is in general performed over replicate experiments and for the reported log-MSE values also over prediction time.

**Table 4:** Performance assessments of predictive monitoring of DO values.

Time [%]	log(MSE${}_{\mathrm{PF}})$	log(MSE${}_{\mathrm{SM}})$	% sig	td${}_{\mathrm{PF}}$ [h]	td${}_{\mathrm{SM}}$ [h]	Loss${}_{\mathrm{PF}}$ [%]	Loss${}_{\mathrm{SM}}$ [%]
Gompertz growth model on LB-500 mL							
30	−1.42	−4.17	0.00	0.78	0.05	−3.76	−5.85
40	−2.93	−4.01	0.00	0.10	0.05	−2.38	−5.85
50	−3.44	−3.99	0.00	0.10	0.05	−13.19	−5.85
60	−1.96	−4.34	0.00	0.39	0.05	−19.94	−5.85
Gompertz growth model on TB-500 mL							
30	−2.30	−4.21	0.00	−0.20	0.07	37.57	−8.80
40	−3.61	−4.12	0.00	0.00	0.07	6.66	−8.80
50	−3.50	−4.05	0.00	0.17	0.07	−20.35	−8.80
60	−5.28	−6.69	16.67	0.12	0.07	−18.45	−8.80
Logistic growth model on LB-500 mL							
30	−2.21	−3.79	0.00	0.36	0.06	−18.31	−7.27
40	−2.67	−3.62	0.00	0.06	0.06	−2.17	−7.27
50	−3.00	−3.64	0.00	0.21	0.06	−20.51	−7.27
60	−1.53	−4.00	0.00	0.58	0.06	−20.00	−7.27
Logistic growth model on TB-500 mL							
30	−2.40	−3.75	0.00	−0.08	0.08	21.82	−11.33
40	−3.09	−3.68	0.00	0.23	0.08	−20.65	−11.33
50	−2.59	−3.63	0.00	0.33	0.08	−19.66	−11.33
60	−3.68	−5.11	0.00	0.21	0.08	−20.75	−11.33

Column time [%] denotes the time which splits training and test samples in percent of the duration of the entire growth experiment. Columns log(MSE${}_{\mathrm{PF}})$ and log(MSE${}_{\mathrm{SM}})$ denote the average logarithm of the mean square prediction error for the particle filter (index PF) and for the statically fit model (index SM). The use of logarithms improves resolution and implies that smaller values correspond to better performance. Column % sig summarizes the *p*-value calculations in Section 2.9 by reporting the fraction of cultures for which the PF leads to a significant improvement over the SM. Columns td${}_{\mathrm{PF}}$ [h] and td${}_{\mathrm{SM}}$ [h] are calculated according to Equation (4) and report the differences between the predicted time when we expect to reach an oxygen level of 20% of the maximum and the post hoc identified value in hours. The implications of differences between the predicted and the theoretical optimum are summarized in columns loss${}_{\mathrm{PF}}$ [%] and loss${}_{\mathrm{SM}}$ [%]. Averaging is in general performed over replicate experiments and for the reported log-MSE values also over prediction time.

## Data Availability

All data is available as supplementary material.

## Supplementary Materials

The following are available online at https://www.mdpi.com/article/10.3390/bioengineering8110177/s1, File S1: supplementary methods with implementation details of the particle filter for inferring Gompertz and Logistic growth models. File S2: annotation of all data files, BM values and DO concentrations over process time.

## Abbreviations

The following abbreviations are used in this manuscript:

BM	Biomass
DAG	Directed acyclic graph
DO	Dissolved oxygen
EKF	Extended Kalman filter
FDA	Food and drug administration
FDR	False discovery rate
GPL	GNU public license
KF	Kalman filter
LB	Lysogeny broth

ML	Machine learning
mse	Mean square error
MCMC	Markov Chain Monte Carlo
O_2_	Oxygen
OD${}_{600}$	Optical density at 600 nm
PAT	Process analytical technology
PF	Particle filter
QbD	Quality by design
SF	Static fit
SFR	shake flask reader
ssd	Sum of squared differences
TB	Terrific broth
